# Mitral valve re-repair vs replacement following failed initial repair: a systematic review and meta-analysis

**DOI:** 10.1186/s13019-020-01344-3

**Published:** 2020-10-07

**Authors:** Muthu Veerappan, Prashasth Cheekoty, Faizus Sazzad, Theo Kofidis

**Affiliations:** 1 Department of Cardiac, Thoracic and Vascular Surgery, National University Heart Centre, National University Health System-NUHS, 1E Kent Ridge Road, 9th Floor, Tower Block, Singapore, 119228 Singapore; 2 School of Medicine, University of Dundee, Dundee, UK; 3 School of Medicine, Newcastle University, Newcastle, UK; 4 Department of Surgery, Yong Loo Lin School of Medicine, National University of Singapore, Singapore, Singapore; 5 Cardiovascular Research Institute, National University of Singapore, Singapore, Singapore

**Keywords:** Meta-analysis, Mitral valve, Re-repair, Replacement

## Abstract

**Background:**

The optimal treatment strategy following a failed mitral valve repair remains unclear. This study aims to compare and analyse available studies which report the clinical outcomes post mitral valve re-repair (MVr) or replacement (MVR) after a prior mitral valve repair.

**Methods:**

Based on PRISMA guidelines, a literature search was performed utilising PubMed, Cochrane and Scopus databases to identify retrospective cohort studies that reported outcomes of MVr and MVR after a prior mitral valve repair. Data regarding operative mortality, clinical outcomes and complications were extracted, synthesized and meta-analysed where appropriate.

**Results:**

Eight studies with a total cohort of 1632 patients were used. After analysis, no significant differences in the short term and long-term operative mortality, incidence of stroke, congestive heart failure, Grade 1 and Grade 2 mitral regurgitation, requirement of 3rd mitral valve operation and reoperation due bleeding were found between the two groups. However, a slightly higher incidence of postoperative atrial fibrillation (OR: 0.11, CI: 0.02 to 0.17, I^2^ = 0%, *p* = 0.02) was observed in the MVR group, as compared to the MVr group.

**Conclusion:**

MVr appears to be a viable alternative to MVR for mitral valve reoperation, given that they are associated with similar post-operative outcomes.

## Introduction

Mitral regurgitation is the second most frequent indication for valve surgery in industrialised countries, with an overall prevalence of 9.3% in the general population who are above 75 years old [1]. Traditionally, mitral valve replacement was the preferred option of treatment among surgeons as the valve repair was technically more demanding [2]. However, there has been a surge in development of repair techniques post introduction of Carpentier’s techniques [3]. Numerous qualified centres have reported excellent outcomes after mitral valve repair thanks to its reproducible clinical results [4–7]. Therefore, mitral valve repair has been identified as the optimal intervention strategy to correct significant mitral regurgitation [8]. However, the incidence of reoperation after initial mitral valve repair failure estimates at 4.5 to 8% at 10 years [7]. The causes of incomplete repair can be categorised into procedure related and valve related factors [9]. Procedure related factors encompass the technical failures of repair while valve related factors include the progression of native disease or new pathology such as degenerative, rheumatic, ischemic and endocarditis.

Although the incidence and causes of repair failure is well documented, little is known regarding the clinical outcomes post mitral valve re-repair (MVr) or mitral valve replacement (MVR). Thus, the optimal treatment strategy for an initial failed mitral valve repair remains unclear, thereby clouding surgeons’ decision making.

Hence, our present study aims to compare and analyse all available studies which report the clinical outcomes post MVr and MVR after a prior mitral valve repair.

## Materials and methods

### Search strategy

A literature search was performed using the Preferred Reporting Items for Systematic Reviews and Meta-Analyses (PRISMA) guidelines electronically utilizing Medline (PubMed), Cochrane and Scopus databases, from inception to 1st January 2020. A repetitive and exhaustive combination of the following ‘Medical Subject Headings’ (MeSH) were used: ‘mitral valve/surgery’, ‘mitral valve insufficiency’, ‘reoperation’, ‘recurrence’, ‘treatment outcome’, ‘treatment failure’, ‘survival analysis’ and ‘survival rate’. This study protocol was registered with PROSPERO #CRD42020160343. The full search strategy can be found in the supplementary materials (supplementary Table 1). Relevant articles were screened and systematically assessed with inclusion and exclusion criteria applied.

### Eligibility criteria

The inclusion criteria included any retrospective cohort studies in which patients underwent surgical intervention with either a second repair (re-repair) or replacement after a prior mitral valve repair. Only studies that had a clear comparative data differentiation between the re-repair and replacement group were included, with the exception of studies with essential qualitative elements. Furthermore, only studies published after the year 2005 were included to prevent using outdated data. Additionally, any studies that were not written in the English language were excluded.

### Data extraction and outcomes

Three reviewers (P.C, M. V, F.S) screened and assessed the studies independently for inclusion. The scientific papers were first screened by their titles and abstracts, where criteria were purposely broad to include all relevant studies. The full text review was performed on articles if the reviewer was unable to confirm the relevance of the study for inclusion.

Two authors (P.C, M.V) independently abstracted the details of the study population. The preoperative baseline characteristics extracted included the following: mean age, sex, history of diabetes, renal failure, prior atrial fibrillation and left ejection fraction.

Furthermore, relevant data of clinical outcomes was obtained from each study required for the generation of forest plots. Post-operative complications (such as stroke, atrial fibrillation, mitral regurgitation and congestive heart failure), requirement of 3rd mitral valve operation, re-operation due to bleeding, short term and long-term operative mortality were the main outcomes analysed in this study. Short term operative mortality was defined within a 30-day period. In addition, the meta-analysis included the Kaplan-Meier survival rates at 1,5,7 and 10 years.

### Statistical analysis

The meta-analysis of the eligible studies was performed in line with recommendations from the Cochrane Community and the forest plots were generated through the means of Review Manager version 5.3 software (RevMan 5) [10]. Since the clinical outcomes derived from the scientific journals were categorised under continuous data, the effect measures were estimated using odds ratio (OR). Odds ratio which was calculated using the Mantel-Haenszel method represents the odds of an adverse outcome occurring in the MVr compared to the MVR group. Heterogeneity (I^2^) was graded as low (I^2^ < 25%), moderate (25 < I^2^ < 75%), or high (I^2^ > 75%). All meta-analyses were carried out using random-effects models to account for statistical variability across the studies.

Furthermore, in presence of I^2^ > 25%, stability of pooled meta-analyses results were examined by the standard leave-one-out sensitivity analysis. This was conducted by removing the included studies one after another to validate the robustness of the results.

### Quality of evidence and risk of Bias assessment

The 8 included retrospective cohort studies [7, 9, 11–16] was all single centre studies (Table 1). The quality of these studies was assessed using Newcastle-Ottawa Scale [17], seen in Table 2. Studies with a score more than or equal to 6 was considered to be of acceptable quality and included [18].

**Table 1:** Summary of Included Studies

Author	Year	Type	No. of patients	MVr (n)	MVR (n)	Place of Study	Outcome accessed
Nishida et al. [7]	2018	Retrospective cohort	86	23	63	Sakakibara Heart Institute	Operative mortality, postoperative morbidities, Long-term survival (mean follow-up period of 76.3 ± 55.0 months)
Zegdi (Late) et al. [9]	2008	Retrospective cohort	13	9	4	Service de Chirurgie Cardiovasculaire, Paris, France	Mechanisms of late failure, Long-term results were assessed on the basis of NYHA functional class, electrocardiogram and echocardiography
Zegdi et al. [11]	2008	Retrospective cohort	43	21	22	Service de Chirurgie Cardiovasculaire, Paris, France	Feasibility of Redo Mitral Valve Repair, Mechanisms of late Failure, Operative Mortality and Morbidity and Long-Term Outcomes.
Kwedar et al. [12]	2017	Retrospective cohort	812	130	682	US (Medicare Database)	Characteristics of Reoperation Cohort, Outcomes of Reoperation, Time to Reoperation, Hospital Mortality According to Hospital Annual Mitral Procedure Volume and Long-Term Survival
Ma et al. [13]	2018	Retrospective cohort	40	23	17	Shanghai Chest Hospital	Early mortality, Early major morbidities, Survival, reoperation for recurrent mitral valve pathology and echocardiographic data
Kilic et al. [14]	2018	Retrospective cohort	305	48	257	University of Pennsylvania Health System	The primary outcome was operative mortality. Secondary outcome included postoperative complications and long term freedom from death.
Suri et al. [15]	2006	Retrospective cohort	145	64	81	Mayo Clinic Rochester	Indications for Reoperation, Predictors of Late Mortality, Predictors of Third Mitral Operation and Follow up data
Dumont et al. [16]	2007	Retrospective cohort	188	68	120	The Cleveland Clinic	Mechanisms and Timing of Repair Failure, Freedom from reoperation, Incremental Risk Factors for Death after Mitral Valve Reoperation

**Table 2:** Risk of Bias of Retrospective Cohort studies according to the Newcastle-Ottawa Scale

Author (Year)	Selection				Comparability	Outcome			Total Score
	1	2	3	4	5	6	7	8	
Nishida et al. [7]	*	*	*	*	–	*	*	*	6
Zegdi (Late) et al. [9]	*	*	*	*	–	*	*	*	6
Zegdi et al. [11]	*	*	*	*	–	*	*	*	6
Kwedar et al. [12]	*	*	*	*	–	*	*	*	6
Ma et al. [13]	*	*	*	*	–	*	*	*	6
Kilic et al. [14]	*	*	*	*	–	*	*	*	6
Gillinov et al. [15]	*	*	*	*	–	*	*	*	6
Suri et al. [16]	*	*	*	*	–	*	*	*	6
Dumont et al. [17]	*	*	*	*	–	*	*	*	6

As recommended by chapter 14 of the online Cochrane Handbook version 5.1, the software GRADEprofiler (GRADEpro) was further utilised to validate the quality of evidence of the included retrospective studies (Supplementary Table 2) [19]. Apart from the high risk of bias in confounding factors and patient selection that are typical of studies with retrospective nature, we determined that evidence provided by these studies are still of acceptable quality.

## Results

### Included studies and patients

The initial systematic broad search in the PubMed, Cochrane and Scopus databases, using the search strategies highlighted earlier, revealed a total of 1415 papers. Post removal of duplicates using the EndNote X7 reference management software, 1393 papers remained for further review. Based on title and abstract screening irrelevant studies that did not satisfy our inclusion criteria were excluded. The remaining 21 studies were then retrieved for assessment of full text. Twelve studies that either lacked data on the required outcomes of our meta-analysis, or that did not use native data were excluded. A further 1 study was also excluded as it was published before 2005. The remaining 8 eligible studies [7, 9, 11–16] were finalised for the purpose of our meta-analyses on 1st January 2020.

As discussed earlier all the included scientific papers were observational studies that performed a retrospective data collection with an aim to determine whether the MVr or MVR group promised better clinical outcomes for patients.

**Fig. 1:**
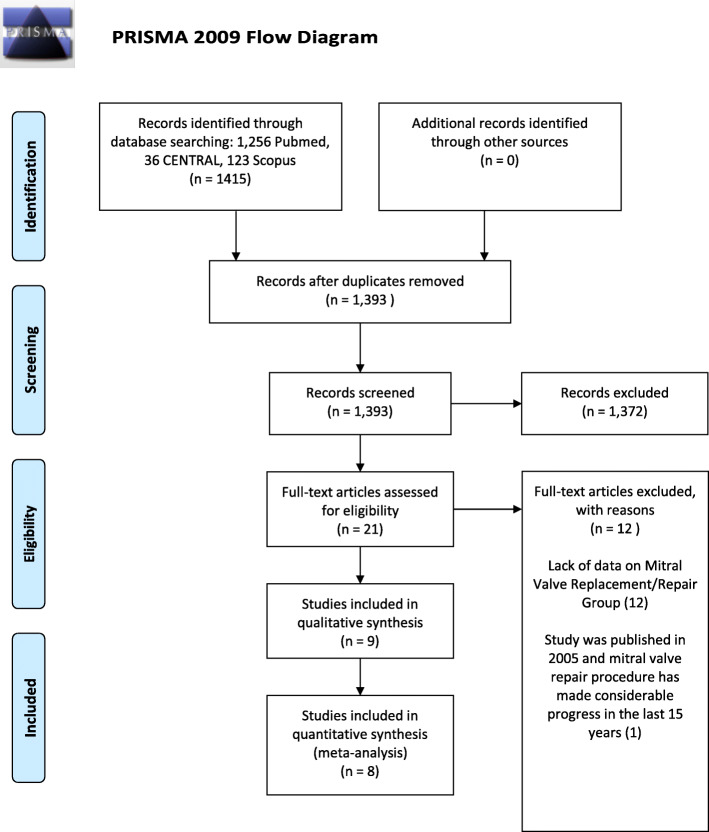
PRISMA Flow Diagram. The systematic search revealed a total of 1415 papers, of which 1393 remained for review after duplicates were removed. After implementation of inclusion and exclusion criteria, 21 articles were selected for full-text review. Following the full-text assessment of these articles, one old study (2005) and studies that lacked data on mitral valve replacement or repair group (*n* = 13) were excluded, leaving 8 papers for inclusion into the present study

The PRISMA statement flowchart shown in Fig. 1 highlights the aforementioned screening process. Furthermore, we are aware that 2 of the 8 included studies [9, 11] in our meta-analysis have been published by the same author, *Zegdi,* in the same year (2008) as seen in Table 1. Assessment of the full texts verified that these studies were performed on two completely different study population, therefore can be included separately in our meta-analysis. To aid the identification of these papers, we used the naming *Zegdi (Late)* et al [9] and *Zegdi* et al [11] to differentiate them.

### Study characteristics

The preoperative baseline characteristics of the study population are summarised in Table 3. Preoperative variables included demographics such as mean age, sex, comorbidities including diabetes mellitus, renal failure, atrial fibrillation and left ventricular ejection fraction.

**Table 3:** Preoperative Baseline Characteristics

Study	Mean Age		Male (%)		Diabetes (%)		Renal Failure (%)		A Fib (%)		LVEF (%)		Valve Related Failure (%)		Procedure Related Failure (%)	
	MVr	MVR	MVr	MVR	MVr	MVR	MVr	MVR	MVr	MVR	MVr	MVR	MVr	MVR	MVr	MVR
Nishida et al**.** [7]	48.7 ± 2.8	62.9 ± 1.7	64		–	–	–	–	31		–	–	61	10	35	84
Zegdi (Late) et al. * [9]	65		77		–		–		8		71		–	–	–	–
Zegdi et al**.** [11]	55	66	71	77	–	–	–	–	–	–	–	–	–	–	–	–
Kwedar et al. * [12]	74		51		22.7		12.2		68		–		–	–	–	–
Ma et al**.** [13]	55 ± 15.7	62.6 ± 8.4	65	71	13	6	–	–	–	–	62.2 ± 4.5	59.7 ± 6.8	–	–	–	–
Kilic et al**.** [14]	61 ± 16	62 ± 15	62	45	21	20	6	14	–	–	–	–	52.5	70.5	47.5	29.5
Suri et al**.** [15]	64 ± 13	67 ± 11	77	65	–	–	–	–	–	–	–	–	44	69	56	31
Dumont et al**.** [16]	57 ± 9.8	63 ± 12	73	57	–	–	–	–	–	–	12	31	–	–	–	–

*For studies including *Kwedar* et al. *and Zegidi (Late)* et al the baseline characteristics at re-operation were not categorised into MVr and MVR

*A Fib* Atrial Fibrillation, *LVEF* Left Ventricular Ejection Fraction

In studies including *Zegdi (Late)* et al. [9] and *Kwedar* et al. [12] the baseline characteristics reported in the study were not categorised into MVr and MVR groups.

The baseline characteristics did not differ markedly between the MVr patients and MVR patients.

Furthermore, the causes of incomplete repair in both the MVr and MVR groups can be categorised into procedure related and valve related factors [9] as seen in Table 3. Procedure related factors encompass the technical failures of repair while valve related factors include the progression of native disease or new pathology such as degenerative, rheumatic, ischemic and endocarditis.

Procedure related factors mainly encompassed ruptures of previously shortened or transferred chordae, elongations or ruptures of chordae from previously shortened papillary muscles, ring dehiscence, leaflet dehiscence or perforations or incomplete repairs [9]. On the other hand, valve related factors included endocarditis, progression of primary disease, valvular prolapse in an area not involved by the previous repair and valvular retractions [9, 14]. A trend was observed where surgeons in *Kilic* et al. [14] and *Suri* et al. [15] studies preferred MVr when the mode of failure was procedure related and MVR when mode of failure was valve related. The contrary was true for *Nishida* et al. study [7].

## Clinical outcomes

### Operative mortality

**Fig. 2:**
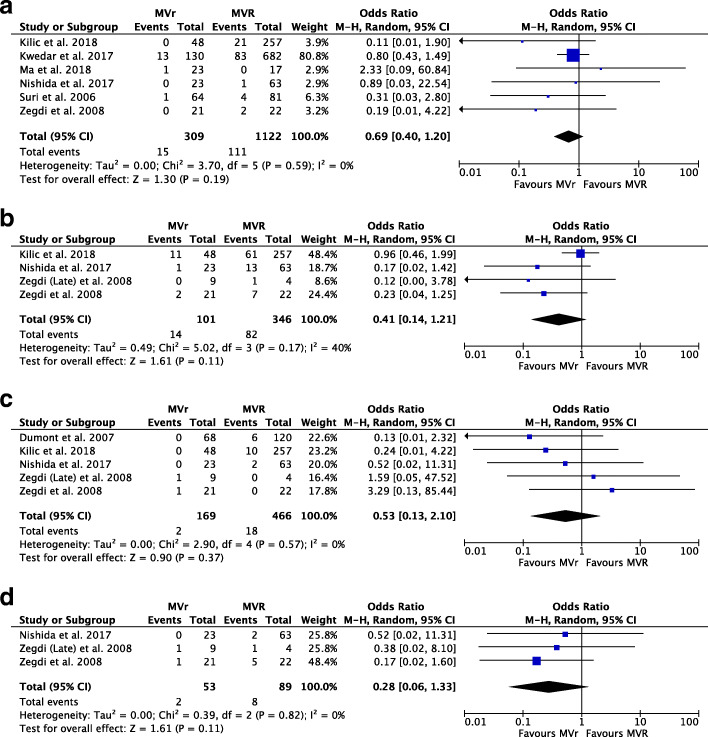
Forest Plots showing (**a**) Short Term Operative Mortality (**b**) Long Term Operative Mortality (**c**) Incidence of Stroke (**d**) Incidence of Congestive Heart Failure. For all these outcomes, we observed no statistical difference between the MVr and MVR groups

From the pooled analysis of 6 studies [7, 11–15], 1431 patients, we observed no statistical difference in short-term operative mortality between the MVr and MVR groups (OR: 0.69, CI: 0.40 to 1.20, I^2^ = 0%, *p* = 0.19) as seen in Fig. 2a.

Similarly, from 4 studies [7, 9, 11, 14], 447 patients, no statistical difference in long-term operative mortality was observed between the 2 groups (OR: 0.41, CI: 0.14 to 1.21, *p* = 0.11) as seen in Fig. 2b. It is important to note that moderate heterogeneity (I^2^ = 40%) was observed. Application of leave-one-out sensitivity analysis (seen in Table 4) revealed that the *Kilic* et al. [14] paper had a substantial influence on the overall significance and heterogeneity of the long-term operative mortality outcome. This heterogeneity could be potentially credited to clinical differences in the *Kilic* et al. [14] paper and its patient population, rather than as a result of statistical heterogeneity across all 4 studies [7, 9, 11, 14]. Therefore, the result of this outcome needs to be interpreted with caution with further exploration of the exact cause of heterogeneity.

**Table 4:** Leave-one-out Sensitivity Analysis: Long-Term Operative Mortality and Requirement of 3rd Mitral Valve Operation

**Study Name**	**MVr vs MVR**		
	**Long-Term Operative Mortality**		
	**OR (95% CI)**	**I**^**2**^ **Statistics (%)**	***p*-value**
Kilic et al. [14]	0.19	0	0.009
Nishida et al. [7]	0.50	41	0.26
Zegdi (Late) et al. [9]	0.45	52	0.19
Zegdi et al. [11]	0.46	42	0.27
	**Requirement of 3rd Mitral Valve Operation**		
	**OR (95% CI)**	**I**^**2**^ **Statistics (%)**	***p*-value**
Dumont et al. [16]	3.87	0	0.04
Kilic et al. [14]	2.01	69	0.47
Suri et al. [15]	0.98	25	0.98
Zegdi et al. [11]	1.62	64	0.58

### Incidence of stroke

Combining the data from 5 studies [7, 9, 11, 14, 16] (Fig. 2c), 635 patients, no significant statistical difference was observed between incidence of stroke in the MVR and MVr groups (OR: 0.53, CI: 0.13 to 2.10, I^2^ = 0%, *p* = 0.37).

### Incidence of congestive heart failure

Pooling the data from 3 studies [7, 9, 11] (Fig. 2d), 142 patients, no significant statistical difference was observed between incidence of Congestive Heart Failure in the MVR and MVr groups (OR: 0.28, CI: 0.06 to 1.33, I^2^ = 0%, *p* = 0.11).

### Requirement of 3rd mitral valve operation

**Fig. 3:**
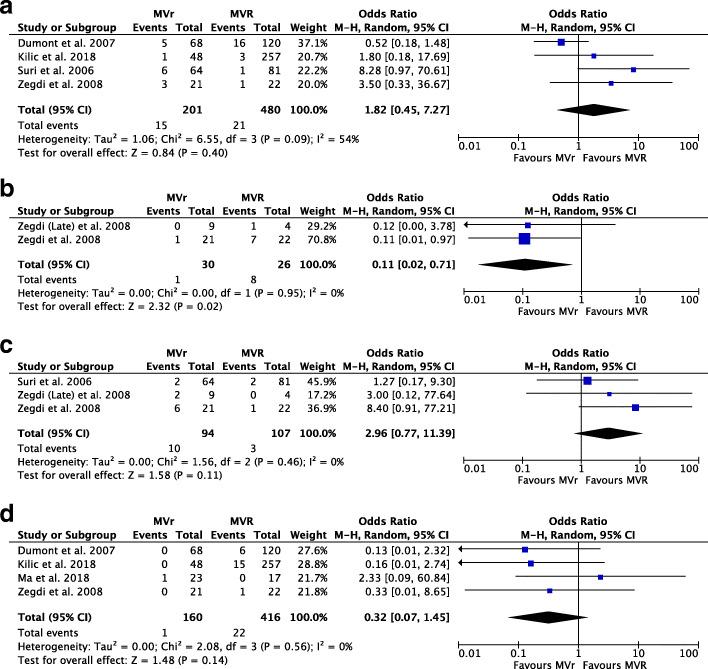
Forest Plots showing (**a**) no observable significant statistical difference in the Requirement of 3rd Mitral Valve Operation (**b**) slightly higher incidence of Atrial Fibrillation in the MVR group and no statistical difference in (**c**) Incidence of Grade 1 (Mild) and Grade (Moderate) Mitral Regurgitation (**d**) Re-operation due to bleeding

From the pooled analysis of 4 studies [11, 14–16], 681 patients, there was no observable significant statistical difference between requirement of 3rd mitral valve operation between the MVR and MVr groups (OR: 1.82, CI: 0.45 to 7.27, *p* = 0.40) as seen in Fig. 3a. However, it is important to note that the heterogeneity was reported to be moderate (I^2^ = 54%). The results of the leave-one-out sensitivity analysis seen in Table 4 revealed that the *Dumont* et al. study [16] had a substantial effect on the overall significance and heterogeneity of the outcome. Therefore, the results of this outcome need to be interpreted with caution, with further exploration of the exact cause of heterogeneity.

### Incidence of atrial fibrillation

Combining the data from 2 studies [9, 11] (Fig. 3b), 56 patients, a slightly higher incidence of patients with atrial fibrillation was observed in the MVR group, as compared to the MVr group (OR: 0.11, CI: 0.02 to 0.17, I^2^ = 0%, *p* = 0.02).

### Incidence of grade 1 (mild) and grade 2 (moderate) mitral regurgitation

Pooling the data from 3 studies [9, 11, 15], 201 patients, there seemed to be no statistical significance in the incidence of Grade 1 and Grade 2 mitral regurgitation between the 2 groups (OR: 2.96, CI: 0.77 to 11.39, I^2^ = 0%, *p* = 0.11) as seen in Fig. 3c.

### Reoperation due to bleeding

Using the data from 4 studies [11, 13, 14, 16], 576 patients, there was no significant statistical difference in the incidence of reoperation due to bleeding in patients who underwent MVR as compared to MVr, illustrated in Fig. 3d (OR: 0.32, CI: 0.07 to 1.45, I^2^ = 0%, *p* = 0.14).

## Discussion

To the best of our knowledge, this is the first systematic review and meta-analysis that compared the clinical outcomes between mitral valve replacement and mitral valve re-repair following failure of initial mitral valve repair surgery. Previously, there have been numerous reviews done on the outcomes of first mitral valve repair versus replacement [4–6, 20] but none on a reoperation procedure involving the mitral valve.

In most aetiologies of initial mitral valve failure, it is commonly agreed by surgeons that mitral valve repair, rather than replacement, is the preferred surgical intervention due to the better reported clinical outcomes and freedom from reoperation and death [4–6, 20]. However, in cases of failed initial mitral valve repair surgeons are often reluctant or hesitant to perform a re-repair owning to the first repair failure, and instead opt for replacement surgery [11]. For instance, the study reports that although the feasibility of re-repair in their centre should have ideally been around 80% following the initial surgery, only 50% of the reoperations were reported to have been re-repair procedures as a result of surgeons’ reluctance to confront the hazards of a second mitral valve repair. Therefore, this meta-analysis was performed with the aim to throw light on and compare the clinical outcomes of MVR and MVr to see if the hazards and complications anticipated by the surgeons in fact hold true and, if not, hopefully provide a more definitive perspective.

The feasibility of mitral valve repair depends on Carpentier’s golden rules: the availability of sufficient leaflet tissue and its pliability [11]. The general preference of repair over replacement is attributed to better preservation of left ventricular function and reduced valve-related complications. Additionally, repair surgeries preserve native valve tissue and avoid the use of chronic anticoagulation therapy, unlike in replacement procedures, favouring better recovery of cardiac function [11]. Hence, this meta-analysis investigates whether these benefits persist after a redo mitral valve repair.

While looking at the clinical outcomes across the two groups, there were no significant statistical differences for incidence of stroke, congestive heart failure, requirement of 3rd mitral valve operation, mitral regurgitation and reoperation due to bleeding. These findings suggest MVr and MVR are associated with similar postoperative outcomes. Although mitral valve re-repair presented with a lower incidence of atrial fibrillation as compared to the valve replacement group, a larger patient pool needs to be used to substantiate this claim.

Six studies [7, 11–15] reported no significant difference in incidence of short-term operative mortality (as seen in Fig. 2a) between the MVr and MVR groups. Similarly, there was no significant statistical difference in the long-term operative mortality (seen in Fig. 2b) between the two groups from 4 studies [7, 9, 11, 14]. Interestingly, the 10 years Kaplan-Meier survival probability (Table 5) post reoperation favoured the mitral valve repair group in *Nishida* et al. [7] and *Zegdi* et al. [11] studies. However, this would require more long-term follow-up data with a larger patient cohort to make more definitive claims.

**Table 5:** Kaplan-Meier Survival Probability Rates

Study	1 Year Survival		5 Year Survival		7 Year Survival		10 Year Survival	
	MVr	MVR	MVr	MVR	MVr	MVR	MVr	MVR
Nishida et al. [7]	100%	94%	100%	82%	100%	82%	100%	82%
Zegdi et al. [11]	95%	87%	95%	75%	95%	69%	95%	65%
Kwedar et al. [12]	76.9%	58.6%	–	–	–	–		
Kilic et al. [14]	96%	86%	78%	68%	–	63%	–	53%
Suri et al. [15]	96%	94%	76%	60%	–	–	–	–

Mitral valve repair technique was dependent on the pathology of the mode of failure. The most common method of correction included triangular or rectangular resection and suture repair of the involved portion of the posterior leaflet supplemented by a standard-length flexible posterior annuloplasty band [7, 16]. Anterior leaflet prolapse was corrected by means of chordal shortening, chordal transfer, or commissural annuloplasty [16]. However, in recent times, surgeons tend to prefer the use of artificial polytetrafluoroethylene neochordae to repair these anterior leaflet lesions [7, 16]. In cases of suture dehiscence or ring detachment, surgeons directly re-sutured if the valve leaflet was pliable [7]. If not pliable, they reinforced it with the autologous pericardium. Edge to edge suturing was used frequently as an additional procedure to correct complicated regurgitation [13].

On the other hand, mitral valve replacement was performed using biological or mechanical valves or mitral homograft. A trend was observed in a number of studies where surgeons preferred biological valves for MVR patients. In *Zegdi (Late)* et al, [9], 50% of the MVR patients had bioprosthesis, 25% had mechanical prostheses and the remaining 25% had mitral homograft. In *Zegdi* et al. [11], 59% of MVR patients had bioprosthesis, 36% had mechanical prostheses and remaining 5% had mitral homograft. In *Kilic* et al. [14], 92% of the MVR patients had mechanical prostheses while the rest had bioprosthesis. In *Dumont* et al. [17], 52% of MVR patients had bioprosthesis and the rest had mechanical prostheses.

### Limitations

Despite the benefits of a pooled analysis, such as higher statistical power, there are several limitations of our current meta-analysis study. Firstly, the retrospective studies included in our meta-analysis carried inherent biases such as selection bias given their observational nature.

The decision to proceed with MVr or MVR depends on various factors including other comorbidities and factors such as age and sex, which lie outside the scope of our univariate analysis. Additionally, some centres might have had monetary considerations/restrictions which would have biased their choice of surgical intervention, along with the surgeon’s experience.

Another limitation we faced was the scarcity of randomised controlled trials available in literature comparing MVr and MVR procedures.

Therefore, further exploration and analysis needs to be performed with a larger patient cohort to provide a more substantial evidence for optimal treatment strategy following a failed mitral valve repair.

## Conclusion

In conclusion, our meta-analysis identified that redo mitral valve repair shares similar post-operative clinical outcomes as compared to replacement surgery. Although surgeons prefer to opt for mitral valve replacement over re-repair, our meta-analysis suggests that the latter might be a viable alternative. However, we recognise the fact that not all patients are suitable candidates for mitral valve re-repair due to the aetiology of their mitral valve failure and other factors such as age and presenting preoperative comorbidities for which reason further exploration has to be done.

## Supplementary information

**Additional file 1: Table S1.** Complete Search Strategy used for PubMed Database. **Table S2.** Summary of Quality of Evidence of Included Studies.

## Data Availability

Not applicable.

## Abbreviations

MVR: Mitral valve replacement
MVr: Mitral valve re-repair
