# Supplementary material for: Mitral valve re-repair vs replacement following failed initial repair: a systematic review and meta-analysis
Source: J Cardiothorac Surg. 2020 Oct 7;15:304. doi: 10.1186/s13019-020-01344-3 (PMC7542900; doi:10.1186/s13019-020-01344-3)
Supplement: Supplementary file 1 — Additional file 1: Table S1. Complete Search Strategy used for PubMed Database. Table S2. Summary of Quality of Evidence of Included Studies. [file 13019_2020_1344_MOESM1_ESM.docx]

**Table S1: Complete Search Strategy used for PubMed Database**

**Phase 1: Search Terms for Procedure**

| **Search #** | **Search Strategy** | **Items Found** |
| --- | --- | --- |
| # 1 | “Mitral Valve/surgery”[Mesh] | 13398 |
| # 2 | “Mitral Valve Insufficiency/surgery”[Mesh] | 10191 |
| # 3 | #1 OR #2 | 19397 |
| # 4 | “Reoperation”[Mesh] | 84486 |
| # 5 | “Recurrence”[Mesh] | 180208 |
| # 6 | #4 OR #5 | 255821 |
| # 7 | #3 AND #6 | 2635 |

**Phase 2: Search Terms for Outcomes**

| # 8 | “Treatment Outcome”[Mesh] | 1020593 |
| --- | --- | --- |
| # 9 | “Treatment Failure”[Mesh] | 34318 |
| # 10 | “Survival Analysis”[Mesh] | 286246 |
| # 11 | “Kaplan-Meier Estimate”[Mesh] | 64163 |
| # 12 | “Survival Rate”[Mesh] | 167942 |
| # 13 | “Retrospective Studies”[Mesh] | 793723 |
| # 14 | #8 OR #9 OR #10 OR #11 OR #12 OR #13 | 1810216 |

**Phase 3: Combining the Phrases**

| # 15 | #7 AND #14 | 1567 |
| --- | --- | --- |

**Phase 4: Limiting the Studies to English**

| # 16 | #15 Filters: English | 1415 |
| --- | --- | --- |

**Table S2: Summary of Quality of Evidence of Included Studies**

| **Certainty assessment** | | | | | | | **№ of patients** | | **Effect** | | **Certainty** | **Importance** |
| --- | --- | --- | --- | --- | --- | --- | --- | --- | --- | --- | --- | --- |
| **№ of studies** | **Study design** | **Risk of bias** | **Inconsistency** | **Indirectness** | **Imprecision** | **Other considerations** | **MVr** | **MVR** | **Relative (95% CI)** | **Absolute (95% CI)** |  |  |
| **Reoperation after mitral valve repair for degenerative disease** | | | | | | | | | | | | |
| 1 | observational studies | serious ^a^ | not serious | not serious | not serious | none | 68/188 (36.2%) | 120/188 (63.8%) | not estimable |  | ⨁◯◯◯ VERY LOW |  |
| **Reoperation for failure of mitral valve repair** | | | | | | | | | | | | |
| 1 | observational studies | serious ^a,b^ | not serious | not serious | not serious | none | 17/81 (21.0%) | 64/81 (79.0%) | not estimable |  | ⨁◯◯◯ VERY LOW |  |
| **Redo mitral valve surgery following prior mitral valve repair** | | | | | | | | | | | | |
| 1 | observational studies | not serious | not serious | not serious | not serious | none | 48/305 (15.7%) | 257/305 (84.3%) | not estimable |  | ⨁⨁◯◯ LOW |  |
| **Outcomes of Early Mitral Valve Reoperation in the Medicare Population** | | | | | | | | | | | | |
| 1 | observational studies | very serious ^a,b,c^ | not serious | not serious | not serious | none | 130/812 (16.0%) | 682/812 (84.0%) | not estimable |  | ⨁◯◯◯ VERY LOW |  |
| **Management of Incomplete Initial Repair in the Treatment of Degenerative Mitral Insufficiency** | | | | | | | | | | | | |
| 1 | observational studies | serious ^a^ | not serious | not serious | not serious | none | 23/40 (57.5%) | 17/40 (42.5%) | not estimable |  | ⨁◯◯◯ VERY LOW |  |
| **Causes of repair failure for degenerative mitral valve disease and reoperation outcomes** | | | | | | | | | | | | |
| 1 | observational studies | serious ^a^ | not serious | not serious | not serious | none | 23/86 (26.7%) | 63/86 (73.3%) | not estimable |  | ⨁◯◯◯ VERY LOW |  |
| **Recurrent mitral regurgitation after repair: should the mitral valve be re-repaired?** | | | | | | | | | | | | |
| 1 | observational studies | serious ^a^ | not serious | not serious | not serious | none | 64/145 (44.1%) | 81/145 (55.9%) | not estimable |  | ⨁◯◯◯ VERY LOW |  |
| **Late posterior failure after mitral valve repair in degenerative disease** | | | | | | | | | | | | |
| 1 | observational studies | serious ^a^ | not serious | not serious | not serious | none | 21/43 (48.8%) | 22/43 (51.2%) | not estimable |  | ⨁◯◯◯ VERY LOW |  |
| **Reoperation for failure of mitral valve repair in degenerative disease: a single-centre experience** | | | | | | | | | | | | |
| 1 | observational studies | serious ^a^ | not serious | not serious | not serious | none | 9/13 (69.2%) | 4/13 (30.8%) | not estimable |  | ⨁◯◯◯ VERY LOW |  |

**CI:** Confidence interval

#### Explanations

a. Confounding and selection bias as the pathology of the patient's diseased mitral valve influenced the surgeon's decision to proceed with re-repair or replacement

b. Bias due to missing data from MVr and MVR group

c. Bias in selection of the reported result as only the data for MVr group was provided/published
